# Hair Cortisol Concentrations as a Biomarker to Predict a Clinical Pregnancy Outcome after an IVF Cycle: A Pilot Feasibility Study

**DOI:** 10.3390/ijerph17093020

**Published:** 2020-04-27

**Authors:** Diana C. Santa-Cruz, Rafael A. Caparros-Gonzalez, Borja Romero-Gonzalez, Maria Isabel Peralta-Ramirez, Raquel Gonzalez-Perez, Juan Antonio García-Velasco

**Affiliations:** 1 Faculty of Health Sciences, Universidad Rey Juan Carlos, Avenida de Atenas, s/n, 28922 Madrid, Spain; juan.garcia.velasco@ivirma.com; 2 IVI-RMA Madrid, Avenida del Talgo, 68−70, 28023 Madrid, Spain; 3 Faculty of Health Sciences, Department of Nursing, University of Granada, 18071 Granada, Spain; rcg477@ugr.es; 4 Mind, Brain and Behavior Research Center (CIMCYC), Faculty of Psychology, University of Granada, 18011 Granada, Spain; borjaromero@ugr.es (B.R.-G.); mperalta@ugr.es (M.I.P.-R.); 5 Faculty of Psychology, Personality, Assessment and Psychological Treatment Department, University of Granada, 18011 Granada, Spain; 6 Department of Pharmacology, CIBERehd, Instituto de Investigación Biosanitaria ibs GRANADA, School of Pharmacy, University of Granada, 18011 Granada, Spain; rgonzalezperez@ugr.es

**Keywords:** infertility, pregnancy, cortisol, stress

## Abstract

Our objective was to examine the feasibility of hair cortisol concentrations (HCC) as a biomarker to predict clinical pregnancy outcomes and investigate its potential associations with perceived anxiety, resilience, and depressive symptoms. A total of 43 participants were assessed using HCC, the state trait anxiety inventory (STAI), resilience scale (RS), and the depression subscale of the symptom checklist 90-R (SCL-90-R). Participants were approached at their second consultation with the reproductive endocrinologist (T1), before scheduling their IVF cycle, and then 12 weeks after (T2), at their post-transfer visit with the study coordinators, before the human chorionic gonadotropin (HCG) pregnancy test. The logistic regression model revealed that HCC at T2 predicted 46% of a positive pregnancy test [R2 = 0.46, (ß = 0.11, *p* < 0.05)]. Pregnant women had higher levels of resilience at T2 (M = 149.29; SD = 17.56) when compared with non-pregnant women at T2 (M = 119.96; SD = 21.71). Significant differences were found between both groups in depression at T2 (t = 3.13, *p* = 0.01) and resilience at T2 (t = −4.89, *p* = 0.01). HCC might be a promising biomarker to calculate the probability of pregnancy in women using assisted reproductive technologies (ART).

## 1. Introduction

Infertility is the inability to achieve a viable pregnancy after one year of unprotected intercourse and is estimated to affect more than 186 million people worldwide [1]. Women take the societal pressure and carry more guilt for infertility even though men are responsible for over half the cases of involuntary childlessness [2]. This can be a contributing factor to the psychological impact that women experience at a greater level than men. The experience of infertility can be considered upsetting on its own, as it includes several elements that can be perceived as stressful: Unpredictability, negativity, uncontrollability, and ambiguity [2].

Women wishing to bear children and experiencing infertility tend to view this condition as unwanted, negative, and threatening. The belief that they have little control over whether they will conceive successfully is common, as is the uncertainty about the odds of reaching their goal [3]. Women may suffer anxiety, and after several unsuccessful rounds of IVF, they may feel sad, angry, and depressed [4,5]. A recent literature review showed that 25% to 40% of infertile couples reported psychopathological symptoms and their anxiety and depression levels were higher than fertile control groups [6].

Resilience can be defined as the ability to elude negative consequences, either social, psychological, or biological, of chronic stress that could compromise an individual’s psychological or physical welfare [7,8]. Resilience can be an essential element of cognitive appraisal: Resilient people feel self-confident they can endure and get over disagreeable states and emotions and, consequently, do not react disproportionately to problems, but rather show composure. In response to setbacks, resilient individuals can focus on other meaningful goals and pursuits, as well as what may reduce their depressive symptoms [9].

Psychological stress, on the other hand, arises from interactions between individuals and their environment that are perceived as straining or that transcend their adaptive capacities and threaten their wellbeing [10]. One of the core neuroendocrine response systems, the hypothalamic-pituitary-adrenocortical (HPA) axis, is highly reactive to emotional stress. Cortisol is the central glucocorticoid in humans, yielding countless affective, immunological, metabolic, cardiovascular, and cognitive effects as well as repercussions on the HPA axis [11,12,13]. Acute stress is usually a very adaptive phenomenon, allowing the individual to fight the stressor and recover. When experiencing chronic stress an individual is at a higher risk for numerous diseases including highly widespread conditions such as anxiety, depression, postpartum depression [13,14], Alzheimer [15], cardiovascular diseases, and metabolic syndrome [16]. This means that if a stressor, such as in infertility, becomes chronic, negative complications can follow. 

Chronic stress can compromise fertility in several ways: It can reduce libido, ovulatory capacity, and implantation success [12]. Other consequences of stress, such as an increase in smoking, substance use, sleep problems, and eating disorders, can further reduce the chances of conceiving following assisted reproductive techniques (ART). Even though the association between stress and impaired fertility has been well-established and explored in non-humans, the question of whether human fertility is directly affected by stress remains a challenge. In the last few years, there was a number of contradictory findings facing research in this field. A few studies showed that the more distressed the woman felt before and during treatment, the lower the possibility of a successful gestation [17,18,19,20,21,22]. Other studies have failed to find any significant differences [23,24,25,26,27]. One of the reasons for the mixed results may be the heterogeneity of methods used to measure stress using different sampling matrices (saliva, blood, urine, and follicular fluid). These methods can only detect cortisol levels for reduced periods of time and therefore cannot measure retrospectively long-term stress [28,29]. Besides, cortisol levels from saliva, blood, urine, or follicular fluid can be easily affected by individual and environmental factors, circadian rhythms, and gonadotrophin protocols [29,30]. However, in the last few years, encouraging findings have become available concerning multiple essential features of hair cortisol concentrations (HCC), including its validity as a measure of long-term systemic cortisol concentrations, its reliability across repeated analysis, and its relative robustness to a variety of potential confounding factors. This makes HCC a valuable biological marker to assess chronic stress. In contrast to other sampling matrices, HCC offer a retrospective assessment of accumulated exposure to stress, can be collected using non-invasive procedures, and stay unaffected by circadian rhythmicity. To date, only two studies have investigated the association between HCC and ART [14,22] and one of them found that HCC predicted clinical pregnancy [22]. 

The aim of this pilot study is threefold. First, we aimed to build upon previous research by determining the feasibility of HCC as a biomarker with predictive value regarding treatment outcome. Second, we aimed to explore cortisol’s relationship with perceived anxiety and resilience levels as well as depressive symptoms, documenting the general pattern of stress during two time points and its correlation to clinical pregnancy. The third goal was to explore the viability of our study design as a first step in designing a new study protocol to address chronic stress during ART. 

## 2. Materials and Methods 

**Figure 1:**
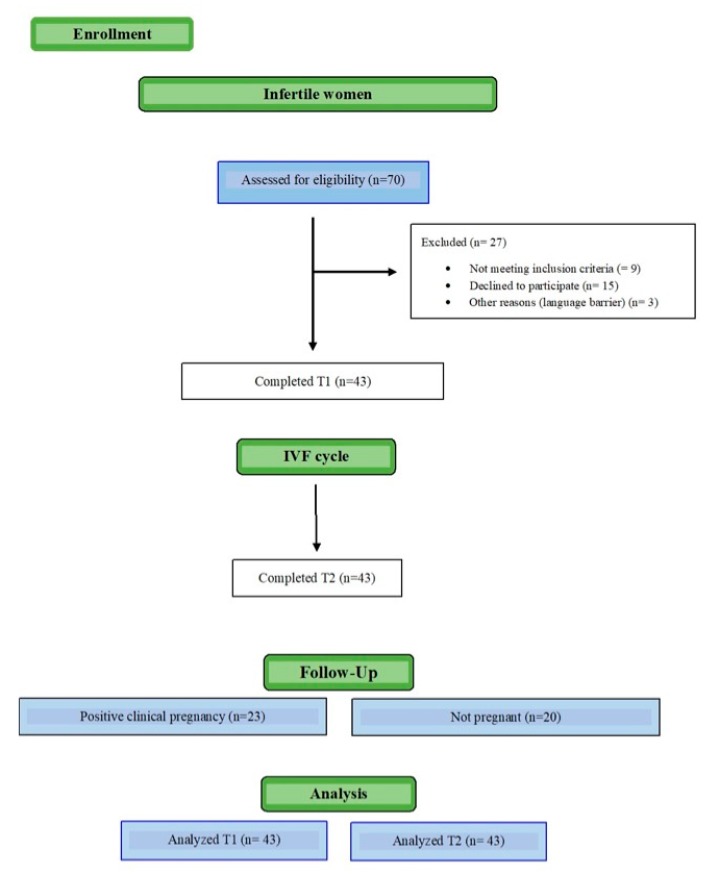
Participant flow diagram.

A total of 70 women were approached, 43 of whom gave written consent to participate, left an initial sample of hair, and completed baseline psychological assessment (Figure 1). Of the 43 women enrolled at T1, 43 participants were assessed through questionnaires and HCC at T2.

Inclusion criteria were, women with a body mass index of 19–30 kg/m^2^, no previous fertility treatments, undergoing in vitro fertilization with intracytoplasmatic sperm injection (IVF/ICSI) or in vitro fertilization with preimplantation genetic diagnosis (IVF/PGD), with delayed embryo transfer under the gonadotropin-releasing hormone (GnRH) antagonist protocol at a university affiliated private fertility clinic. In the antagonist cycle, luteinizing hormone (LH) suppression was accomplished by subcutaneous (SC) injections of 0.25 mg of GnRH antagonist starting in the presence of follicles >14 mm or E2 levels >400 pg/mL and continuing until ovulation triggering. To keep the sample as homogeneous as possible, study exclusion criteria were: Subjects with any recognized psychiatric (e.g., psychotic/bipolar disorder or major depression) or an immune health condition like lupus or rheumatoid arthritis; previous fertility cycles; previous children; no fluency in Spanish; and drugs, alcohol, or high caffeine consumption (>4 cups of coffee). To reduce the confounding bias effect of corticoid medications in cortisol levels, patients under steroid medication or diagnosed with Cushing disease, asthma, diabetes, or other diseases known to impact cortisol levels, were excluded. Data were collected during the study period (June 2019 to September 2019). All women read and signed an informed consent form. This study followed the Helsinki Declaration (AMM, 2008), Good Clinical Practice Directive (Directive 2005/28/EC) of the European Union, and was authorized by the Ethics Committee, reference number 1405-MAD-027-DS.

Participants agreeing to participate were informed of the study characteristics by the study coordinators, and were assessed using HCC, and psychological assessment by means of the resilience scale (RS), state trait anxiety inventory (STAI), and the symptom checklist 90-R (SCL-90-R) (described in the psychological assessment section). Participants were approached at their second consultation with the reproductive endocrinologist (T1) before scheduling their first IVF cycle, and then 12 weeks after at their post-transfer visit with the study coordinators (T2), days before human chorionic gonadotropin pregnancy test. All patients underwent a frozen-thawed delayed embryo transfer and none were aware of treatment outcome at T2. All patients underwent a transfer of a single embryo with good quality, blastocyst stage. 

### 2.1. Sociodemographic and Clinical Data

The sociodemographic and clinical details were collected from the medical file of the patients. Data from the baseline self-report questionnaires and the clinical information were united in order to have the most comprehensive information possible for age, medical diagnosis of infertility (male factor, female factor, or mixed factor), duration of infertility, and miscarriages prior to the first IVF cycle. Physical activity level was divided into low (no physical exercise), moderate (1–2 times weekly), and high (more than 3 times weekly). Three months after T2, medical parameters were obtained from the database, including ongoing clinical pregnancy, detected by ultrasound visualization of fetal heart beat at 6 weeks after the embryo transfer [22].

### 2.2. Hypothalamic-Pituitary-Adrenal Axis Activation Assessment: Hair Cortisol Concentrations

In order to evaluate the activation of the HPA axis, HCC levels were assessed using hair samples proximal to the scalp with a minimum length of 3 cm (cumulative cortisol deposited over the previous 3 months considering that hair grows 1 cm per month). Hair samples were cut with scissors from the head. Then, hair samples were placed in a piece of aluminum foil so that light and humidity will not damage it. Afterwards, it was stored in a dry and dark cupboard at the laboratory. The samples were then analyzed at the Faculty of Pharmacy at the University of Granada. Once there, hair samples was transformed to a fine powder using a ball mill [31]. Cortisol from hair samples was obtained into HPLC-grade methanol while incubating the samples for 3 days at room temperature. Supernatant, after incubation, was evaporated dry with a evaporator. The samples were then frozen at −20 °C until they were analyzed.

HCC was measured with a salivary ELISA cortisol kit. This method is a validated to evaluate HCC and is positively associated to liquid chromatograph-mass spectrometry (LC-MS/MS). The sensitivity value of the cortisol kit was 1.0 ng/mL. The cross reactivity was: Prednisolone 13.6%, corticosterone 7.6%, deoxycorticosterone 7.2%, progesterone 7.2%, cortisone 6.2%, deoxycortisol 5.6%, prednisone 5.6%, and dexamethasone 1.6%. HCC were expressed in pg/mg.

### 2.3. Psychological Assessment: Anxiety, Depression, and Resilience 

The Spanish version of the STAI was utilized to assess state and trait anxiety [32,33]. The STAI consists of 2 subscales with 20 items each within a questionnaire. The first subscale, the state anxiety scale (STAI-S) assessed the current state of anxiety by asking how participants felt “right now,” using items that measured personal feelings of worry, nervousness, as well as arousal of the autonomic nervous system. The trait anxiety scale (STAI-T) assessed fairly stable aspects of “anxiety proneness,” including common states of security, confidence, and calmness. All items were rated on a 4-point Likert scale (from “Almost Never” to “Almost Always”). This measure has been shown to have high reliability α = 0.93. We chose the STAI questionnaire to assess anxiety because it is a well-established scale that has been used widely in the population and is sensitive to short-term change.

The Spanish Version of the SCL-90-R was used to measure psychopathological symptoms [34,35]. This 90-item scale is scored using a 5-point Likert scale (from 0 (never) to 4 (extremely)). This instrument is used to evaluate 9 dimensions: Somatization, obsession-compulsion, interpersonal sensitivity, depression, anxiety, hostility, phobic anxiety, paranoid ideation, and psychoticism. When assessing depressive symptoms, we used only the depression scale (DEP). The Spanish version of Cronbach’s alpha reliability coefficient depression subscale range was between 0.87 and 0.94 [36].

The RS is comprised of a 17-item “Personal Competence” subscale and an 8-item “Acceptance of Self and Life” subscale [37,38]. Resilience as described by Wagnild and Young consists of 5 fundamental characteristics: Meaningful life (purpose), perseverance, self-reliance, equanimity, and existential aloneness. The Chronbach’s alpha reliability coefficient of the Spanish version is similar to other populations (α = 0.79). 

### 2.4. Statistical Analysis

We conducted all statistical analyses using SPSS version 22.0 for Mac (SPSS, Armonk, NY, USA). A multiple imputation procedure was done to analyze the trend of the missing data. The imputation method employed was the fully conditional specification. This is an iterative Marco chain Monte Carlo (MCMC) method which may be used when an arbitrary pattern of missing data is identified. 

Data screening was performed to ensure that the data fit the assumptions of parametric testing including homogeneity and homoscedasticity of variances and normal distribution. All the outliers greater than +3 or less than −3 SD were removed before the analysis. A score of R40 on the STAI-S and STAI-T was used to detect clinically significant anxiety. On the RS we used a score above 170 as “good resilience”. SCL-90-R scores of DEP 2.0 (or >70 for clinical scores) were used to detect depression symptoms. Descriptive analysis was conducted employing arithmetic mean and standard deviation for quantitative variables, and absolute frequency and percentage for categorical variables. Potential associations between variables were measured using Pearson’s correlation analyses. Since the scores for HCC did not present a normal distribution we performed a natural log transformation (LN_T1 and LN_T2) of HCC to improve the distribution characteristics and reduce skewness. This procedure was followed successfully in previous studies [14]. To analyze whether the main sociodemographic, cortisol, and psychological variables were equally distributed in both groups (pregnant and not pregnant), we performed several Student’s t-test and chi-squared test analyses. To evaluate whether the two groups showed significant differences for psychological variables, we conducted several ANOVA. Logistic regression through analysis of maximum likelihood estimates were calculated to assess the influence of HCC and psychological symptoms (depression, resilience, and depression) on a positive pregnancy test.

Following G*Power Statistical software, in order to achieve optimal results, sample size requirements was established at a minimum sample size of 43 to acquire an adequate power of 0.8, applying an α = 0.05 [39].

## 3. Results

The mean age of the participants was 36.3 years (SD = 4.36). All participants were in a heterosexual relationship and married. None of them was receiving psychological therapy or were on psychiatric medication at the time of the treatment. Most of the participants were educated at a high school level (n = 23) and some graduated university (n = 18). The average duration of infertility was 18.3 months (SD = 8.6). Over half of the sample (n = 23) achieved clinical pregnancy after their first IVF/ICSI or IVF/PGD cycle. None of the sociodemographic variables changed from T1 to T2. The sociodemographic, lifestyle, and clinical variables are shown in Table 1.

Table 2 shows the differences between pregnant women and non-pregnant women in respect to HCC, anxiety, depression, and resilience. No differences were found regarding HCC and anxiety between the group of pregnant women and the group of non-pregnant women. 

### 3.1. Stress Assessment through a Biomarker: Hair Cortisol Concentrations 

**Figure 2:**
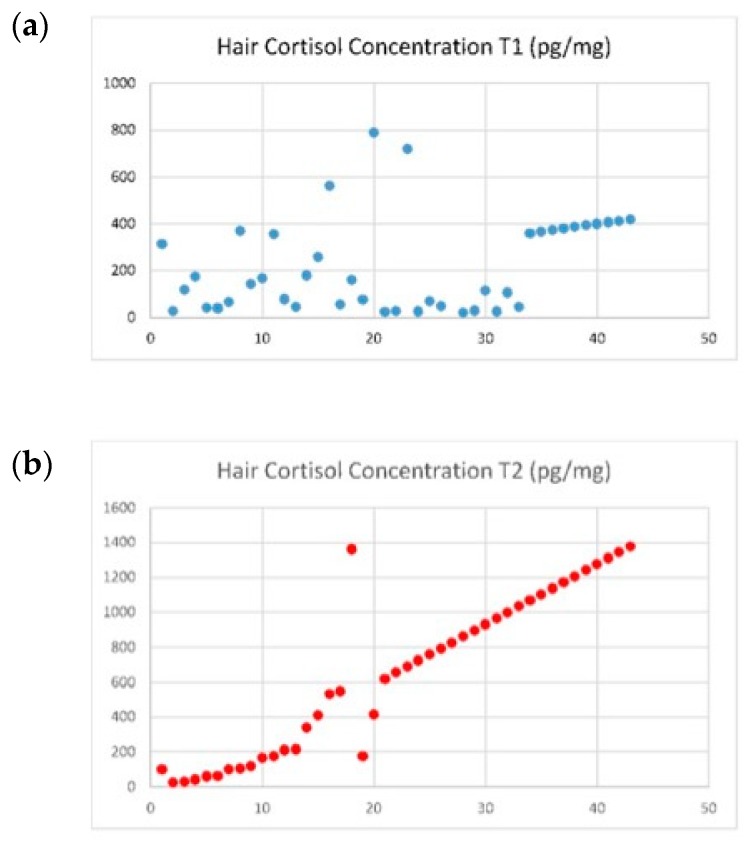
Evolution of HCC for the total sample. (**a**) Evolution of HCC in T1 for the total sample; (**b**) Evolution of HCC in T2 for the total sample.

Hair cortisol concentrations in the total sample increased from T1 to T2 (279.25 vs. 655.92 pg/mg), t = 4.17, *p* < 0.001) (Table 2 and Figure 2). On T2, 23 women (53.5%) had a positive pregnancy test, and their HCC increased from T1 to T2 (364.63 vs. 581.90 pg/mg, *p* < 0.001). Women with a negative pregnancy test had higher HCC at T2 (181.06 pg/mg vs. 741.06 pg/mg, *p* < 0.001) but HCC at T2 was not statistically different between pregnant women when compared to non-pregnant women. There were no statistical differences between HCC at T1 and HCC at T2. An interaction effect was found between HCC at T1 and T2 when age, BMI, time of infertility, and number of follicles were included in the model as covariates, F (1,43) = 0.71, *p* = 0.01, and η² = 0.16.

The logistic regression model revealed that HCC at T2 predicted 46% of a positive pregnancy test [R2 = 0.46, (ß = 0.11, *p* < 0.05)].

### 3.2. Psychological Assessment (Anxiety, Depression, and Resilience)

**Figure 3:**
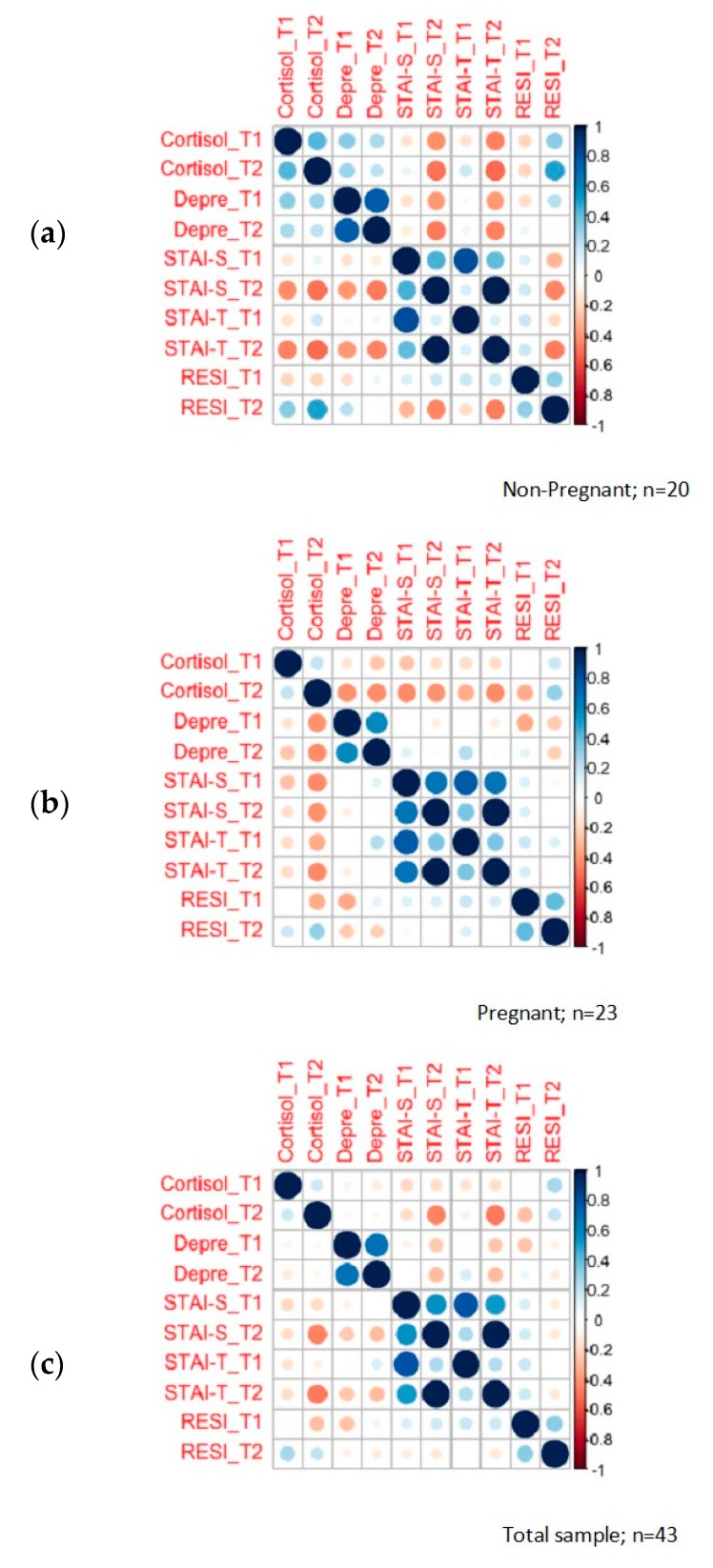
HCC and psychological symptoms correlation plot: (**a**) correlation between psychological symptoms and HCC in non-pregnant women; (**b**) correlation between psychological symptoms and HCC in pregnant women; (**c**) correlation between psychological symptoms and HCC in the total sample.

As shown in Table 2, levels of depression at T2 were higher in the non-pregnant women group (M= 1.33; SD=0.73) in respect to the pregnant women group (M = 0.71; SD = 0.53). Pregnant women had higher levels of resilience at T2 (M = 149.29; SD = 17.56) when compared with non-pregnant women at T2 (M = 119.96; SD = 21.71). Significant differences were found between both groups in depression at T2 (t = 3.13, *p* = 0.01) and resilience at T2 (t = −4.89, *p* = 0.01) as shown in Figure 3. 

The logistic regression model showed that depression at T2 predicted 32% of a positive pregnancy test [R2 = 0.32, (ß = −1.15, *p* < 0.05)]. The logistic regression model result showed that resilience at T2 predicted 34% of a positive pregnancy test [R2 = 0.34, (ß = 0.10, *p* < 0.05)].

## 4. Discussion

We examined whether HCC was a feasible biomarker of chronic stress to be implemented in infertility and whether it was associated to reproductive outcomes. Even though we found no statistical differences in HCC at T2 between pregnant women when compared to non-pregnant women, and no statistical difference between HCC at T1 and HCC at T2, there seems to be a trend that should be confirmed and validated in future studies with a larger sample size. 

We also found that HCC levels at T2 could predict 46% of positive pregnancy, providing some evidence of the potential associations between HCC during an IVF cycle and a positive pregnancy test. Our findings regarding HCC add to a growing body of literature which highlights that chronic cortisol exposure may have significant consequences for health [12,30] and seem to go in line with the findings of another similar study, where associations between hair cortisol and the likelihood of gestation were identified [22].

As far as we know, the present study is the first to explore associations between psychological symptoms, HCC during two time points and the outcome of IVF. Although previous studies have found conflicting results (with some studies finding no change in stress and others finding significant change throughout the IVF cycle) they are very difficult to compare. Many of them used different methodologies, such as obtaining questionnaires and samples (hair, saliva, and follicular fluid) at different time points, as well as recruiting infertile patients who had repeated IVF cycles [17,18,19,20,21,22,23,24,25,26,27]. The time points selected for this study reveal our intention to identify and address anxiety and chronic stress through the IVF cycle rather than focusing on procedure-related anxiety or acute stress [40]. It seems that longitudinal study designs provide a more accurate picture of basal HPA activity as it captures cortisol exposure over a longer period of time and consequently, can easily find associations between stress and likelihood of pregnancy. 

We also found that anxiety, depressive symptoms, and resilience levels changed between T1 and T2, especially in women who had positive pregnancy results. Previous studies showed that the average STAI scores in women undergoing fertility treatments ranged from 33 to 50 [21,41]. In our study, STAI-S mean scores were not significantly elevated throughout fertile population, while hair cortisol levels increased during that timeframe in women with a negative outcome. As it was, in our case, the first time women faced an IVF treatment, their anxiety levels at the initial time point may have been related to the fact that they were facing a new and uncertain, uncontrollable situation. We found that HCC increase and psychological symptoms, both anxiety and depression, at T2 were correlated. These results go in line with previous research done regarding psychological reactions in women in ART [34]. We found that the psychological variables were homogeneous and there were no differences between groups. In our study, depression and resilience at T2 could predict 32% and 34% of clinical pregnancy. Participants with a negative outcome had higher levels of depressive symptoms at T2, before the HCG test result. One possible explanation is that chronic inflammation, recognized as a contributive factor to reproductive dysfunction, can increment depressive symptoms and decrease pregnancy possibilities. Some studies showed that during stress, activation of effector T cells can suppress the development of anxiety and depressive behavior in mice. These repercussions may be intermediated by the interchange of effector T cells to the meningeal space where they generate IL-4, which assists anti-inflammatory responses whilst stimulates the yield of growth factors in the brain that allow neural plasticity and resilience [42,43].

Resilience may boost the confidence needed to appraise chronic stress and therefore, diminish chronic inflammation. The higher the resilience levels, higher the possibility of achieving a positive outcome. Women with lower levels of resilience may feel that they are not able to control their negative emotions [44] and consequently, are not prepared to participate in activities that are intended to reduce these feelings (like physical exercise, balanced diet, quit smoking, and social support) and that could increase their chances of getting pregnant [45]. Furthermore, if levels of resilience are insufficient, women may not be as decided to persevere and go for another IVF cycle, increasing the dropout rate. Consistent with these arguments, psychological resilience-building based cognitive-behavioral therapy should be included during stress-reduction psychological interventions.

A secondary objective of this study was to assess the methodological feasibility prior to designing a new study protocol with a larger sample [46,47]. Our effort to recruit a homogeneous sample of participants with similar estimated pregnancy rates, no previous ART cycles, or any recognized psychiatric or immune condition reduced the number of available sample but allowed us to explore their psychological reactions to the first IVF outcome. Our results validated our initial study design and allowed us to gather important information prior designing a new protocol with a higher number of participants. 

Our findings support the idea that the amount of cortisol in hair could serve as a reliable retrospective biomarker of increased cortisol production during assisted reproductive treatments, measuring exposure to major life stressors and psychological illness with important implications for research and clinical practice.

Although the novelty of this present study will allow for the exploration of new psychological interventions in patients with infertility, we need to acknowledge some limitations. We designed our study as a pilot study, taking into consideration sample size sampling with human samples [47]. In order to validate this procedure, future researchers should try to replicate this study using a larger amount of participants. Larger studies to discover the pathways by which chronic stress hinders fertility are needed [48,49]. The influence of psychological stress through well-validated self-report infertility-stress measures (like the Fertility Problem Stress Scale COMPI-FPSS) along with HCC in a larger sample population [50] should also be addressed. Furthermore, future studies should implement the present protocol using a randomized controlled trial to study the influence of HCC in a positive pregnancy test among women undergoing assisted reproductive technology. When replicating the study, future researchers should control cofounding factors or predictors for pregnancy after IVF/ICSI or IVF/PGD as well as the influence of male partners’ stress levels. Additional biomarkers responsible for the transfer of psychological stress could also be included, such as oxidative stress, cytokines, gut microbiota, serotonin/tryptophan, reactive oxygen species (ROS), and catecholamines [51]. Further research is also needed to analyze the effectiveness of psychological interventions in reducing anxiety, depression [52], and chronic stress and their subsequent impact on IVF treatment outcomes and pregnancy [14,30,53]. 

## 5. Conclusions

HCC is a consistent biomarker to measure chronic stress during ART and predict pregnancy. Assessing stress levels during fertility treatments using psychological questionnaires combined with HCC seemed to yield more exhaustive information, enabling us to gain a better understanding of the associated consequences of chronic stress in fertility and pregnancy outcomes. 

## Figures and Tables

**Table 1:** Sociodemographic and clinical variables.

		Total Sample (n = 43)	Pregnant Women (n = 23)	Non-Pregnant Women (n = 20)	Test *	*p*
Age (Years)		36.20 (±4.63)	36.30 (±4.76)	36.35 (±3.97)	0.034	0.973
Education	High school	25 (58.2%)	12 (52.2%)	13 (65%)	3.08	0.21
	University	18 (41.9%)	11 (47.8%)	7 (35%)		
Physical activity	High	6 (14.0%)	5 (21.7%)	1 (5.0%)	3.81	0.15
	Medium	17 (39.5%)	10 (43.5%)	7 (35.0%)		
	Low	20 (46.5%)	8 (34.8%)	12 (60.0%)		
Body Mass Index (BMI)			23.30 (±2.81)	24.75 (±3.91)	0.104	0.16
Infertility (months)		18.37 (±8.68)	17.30 (±6.10)	19.60 (±10.98)	0.862	0.39
Previous miscarriage (>1)		13 (30.2%)				
Diagnosis infertility	Female factor	20 (46.5%)	11 (47.8%)	9 (45.0%)	2.28	0.32
	Male factor	16 (37.2%)	10 (43.5%)	6 (30.0%)		
	Mixed factor	7 (16.2%)	2 (8.7%)	5 (25.0%)		
Assisted reproductive treatment	FIV/ICSI	20 (46.5%)	12 (52.2%)	8 (40.0%)	0.64	0.42
	FIV/DPI	23 (53.5%)	11 (47.8%)	12 (60%)		
Number of follicles		23 (±4.86)	9.17 (±2.81)	8.40 (±4.57)	−0.51	0.61

Note: * T-test was used to quantitative variables and chi-square test to categorical variables.

**Table 2:** Hair cortisol concentrations and psychological symptoms among pregnant women and non-pregnant women.

	Pregnant Women M (SD) (n = 23)	Non-Pregnant Women M (SD) (n = 20)	T-Student	*p*-Value
**HCC at T1**	364.63 (571.44)	181.06 (169.74)	−1.38	0.17
**HCC at T2**	581.91 (463.91)	741.06 (448.01)	0.47	0.26
**STAI-T at T1**	28.13 (9.11)	26.40 (9.23)	−0.61	0.54
**STAI-T at T2**	28.09 (10.99)	25.95 (10.01)	−0.73	0.46
**STAI-S at T1**	22.39 (10.84)	21.25 (12.11)	−0.32	0.74
**STAI-S at T2**	22.09 (10.99)	21.75 (12.28)	−0.09	0.95
**Depression at T1**	0.78 (0.67)	1.14 (0.82)	1.56	0.12
**Depression at T2**	0.71 (0.53)	1.33 (0.73)	3.13	0.01
**Resilience at T1**	135.89 (15.14)	133.05 (18.31)	−0.55	0.58
**Resilience at T2**	149.29 (17.56)	119.96 (21.71)	−4.89	0.01

Note: HCC—Hair Cortisol Concentrations; STAI-T—Anxiety Trait; STAI-S—Anxiety State.
